# Role of extracellular vesicles in tumour microenvironment

**DOI:** 10.1186/s12964-020-00643-5

**Published:** 2020-10-20

**Authors:** Shi-Cong Tao, Shang-Chun Guo

**Affiliations:** 1 Department of Orthopaedic Surgery, Shanghai Jiao Tong University Affiliated Sixth People’s Hospital, 600 Yishan Road, Shanghai, 200233 China; 2 Institute of Microsurgery on Extremities, Shanghai Jiao Tong University Affiliated Sixth People’s Hospital, 600 Yishan Road, Shanghai, 200233 China

**Keywords:** Extracellular vesicles, Tumour microenvironment, Non-coding RNAs, Lipid biopsy, Drug delivery

## Abstract

In recent years, it has been demonstrated that extracellular vesicles (EVs) can be released by almost all cell types, and detected in most body fluids. In the tumour microenvironment (TME), EVs serve as a transport medium for lipids, proteins, and nucleic acids. EVs participate in various steps involved in the development and progression of malignant tumours by initiating or suppressing various signalling pathways in recipient cells. Although tumour-derived EVs (T-EVs) are known for orchestrating tumour progression via systemic pathways, EVs from non-malignant cells (nmEVs) also contribute substantially to malignant tumour development. Tumour cells and non-malignant cells typically communicate with each other, both determining the progress of the disease. In this review, we summarise the features of both T-EVs and nmEVs, tumour progression, metastasis, and EV-mediated chemoresistance in the TME. The physiological and pathological effects involved include but are not limited to angiogenesis, epithelial–mesenchymal transition (EMT), extracellular matrix (ECM) remodelling, and immune escape. We discuss potential future directions of the clinical application of EVs, including diagnosis (as non-invasive biomarkers via liquid biopsy) and therapeutic treatment. This may include disrupting EV biogenesis and function, thus utilising the features of EVs to repurpose them as a therapeutic tool in immunotherapy and drug delivery systems. We also discuss the overall findings of current studies, identify some outstanding issues requiring resolution, and propose some potential directions for future research.

**Video abstract**

**Supplementary information:**

**Supplementary information** accompanies this paper at 10.1186/s12964-020-00643-5.

## Introduction

**Fig. 1:**
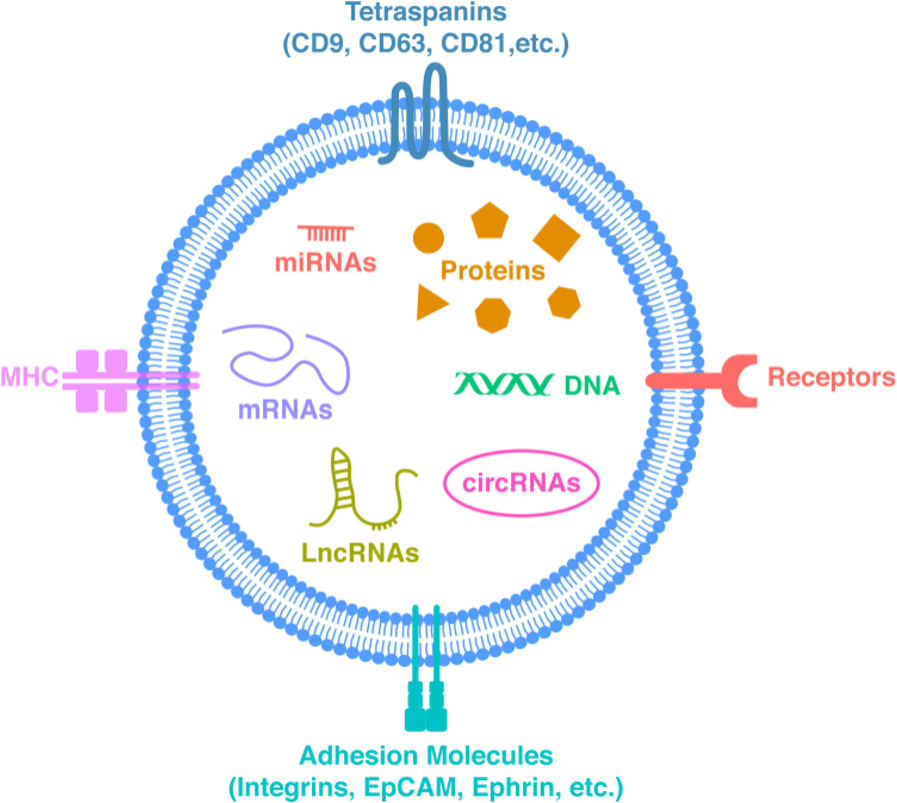
Composition and structure of extracellular vesicles (EVs). EVs is composed of a phospholipid bilayer surrounding protein (membrane protein and cargo protein) and nucleic acid. Membrane proteins include tetraspanins (CD9, CD63, CD81, etc), adhesion molecules (integrins, EpCAM, Ephrin, etc.), MHC, and receptors. Nucleic acid include DNA and RNA (mRNAs, miRNAs, LncRNAs, and circRNAs). Phospholipid bilayer provides protection to the cargo inside

Extracellular vesicles (EVs) are small cell-derived membranous structures, serving as conduits for exchange of significant information between cells [1, 2]. The components of EVs (Fig. 1) include proteins, lipids, messenger RNAs (mRNAs), microRNAs (miRNAs), long non-coding RNAs (LncRNAs), and circular RNAs (circRNAs) [2, 3]. EVs may potentially be the most complex and powerful form of communication in living beings.

EVs have been demonstrated to take part in managing tumour spread and medication resistance [4, 5]. Tumour-derived EVs (T-EVs) negotiate intercellular communication between tumour cells and stromal cells in both regional and distant microenvironments [6]. T-EVs potentially sustain tumour development by regulating several biological functions, including angiogenesis, coagulation, immunity, vascular leakiness, and reprogramming stromal recipient cells to promote pre-metastatic niche (PMN) development and subsequent metastasis [6–9].

Aside from the relatively well-researched proteins, lipids, mRNAs, and miRNAs, emerging evidence demonstrates that LncRNAs and circRNAs participate in managing the microenvironment and tumour progression [10–12]. CircRNAs, a unique class of endogenous non-coding RNAs, can participate in both transcriptional and post-transcriptional regulation. They are characterised by their covalently closed loop frameworks without 5′-caps and 3′-poly tails [13, 14] and operate as reliable miRNA “sponges” (or competing endogenous RNAs; ceRNA) [15, 16], competing with pre-mRNA splicing [17], and participating in circRNA–protein interactions [18].

In this literature review, we provide a brief introduction to EVs and the tumour microenvironment (TME), present findings on the influences of T-EVs on neighbouring cells and the TME, and describe EVs from non-malignant cells (nmEVs) and their influences on the TME. We explore the functions of EVs which are potentially important for the next generation of diagnosis and therapy in the field of malignant tumours, discuss the breakthroughs and shortcomings of current research, and suggest possible future directions of research in this field.

## Tumour microenvironment

The malignant properties of tumours and their advancement are not solely regulated by the tumour cells [19], but also by a variety of non-malignant cell types neighbouring the tumour. These cells in the TME have been identified as essential regulatory agents of tumour promotion [20], and include fibroblasts (FBs), endothelial cells (ECs), adipose cells, mesenchymal stem cells (MSCs), and immune cells [19].

During the onset of tumourigenesis, the microenvironment presents anti-tumour immunity and moderates tumour growth [20], but as the tumour continues to develop, the microenvironment becomes tumour-conducive [20]. The steps involved in this process are of great interest to current researchers. The PMN is defined by the progression of an environment far from the primary tumour, which is appropriate for the survival and outgrowth of any arriving circulating tumour cells [21–23].

The exchange of information in the TME may influence tumour incidence and advancement, in addition to intrusion, metastasis, and various other malignant biological actions [24]. To explain this phenomenon and develop further treatment options, researchers conducted studies based on the traditional/classical theory of intercellular communication. This involves direct contact among cells, as well as paracrine signalling involving cytokines and growth factors between tumour cells and non-malignant cells within the TME [20, 25]. However, there are many unexplained problems with the conventional/traditional theory. Direct contact can only explain the cells that are either already in direct contact or are in direct contact after being recruited. The local effects and effects on recruited cells can only be explained to a certain extent by paracrine factor interaction. Two issues regarding paracrine factors remain: (1) their effects should rapidly decrease with distance; and (2) the complexity of the information that growth factors/cytokines can convey is too small to explain the complex intercellular communication in the TME. Therefore, new theories are required to better explain these features.

## Extracellular vesicles

It has been determined that EVs are intercellular messengers [9, 20, 26, 27]. The lipid bilayer of EVs envelops their components, protecting them from enzymatic degradation [20, 28, 29]. EVs are found in almost all body fluids and are produced by almost all cells, including both eukaryotic and prokaryotic cells [9, 26, 30].

### EV classification

Theoretically speaking, EVs can be classified [30] as either: (1) exosomes (Exos), which are small membrane vesicles (30–100 nm in diameter) derived from the endosome–multi-vesicular bodies (MVBs) pathway; or (2) microvesicles (MVs), which are large membrane vesicles (100–1000 nm diameter) budding away from the plasma membrane. In addition to these classic categories of EVs, apoptotic bodies (a type of large EV, 800–5000 nm in diameter) are shed from apoptotic cells during apoptosis [30]. However, apoptotic bodies scarcely take part in intercellular communication and are widely considered to be eliminated by phagocytes, including macrophagocytes (Mφs), almost immediately after release [30].

However, this classification system is known to be confusing. The definitions of the terms Exos or MVs in the classification criteria are based on EV biogenesis, but in many studies, researchers use these terms based on particle size distributions rather than their true biogenesis. Top-level researchers in the field of EV research, including Clotilde Thery, indicated that despite the fact that Exos and MVs have disparate biogenic mechanisms, the current technology for EV isolation is not able to precisely subdivide these EV sub-populations [30]. Smaller MVs of approximately 100 nm in diameter have likewise been discussed [31]. This term is utilised in numerous studies to describe small EVs recovered by a variety of methods, which do not differentiate endosome-derived EVs (Exos), from plasma membrane-derived EVs (MVs) [6, 32]. Taking this into consideration, with the exception of the section explaining the mechanisms of EV biogenesis, we therefore utilise the term “EVs” rather than “Exos” or “MVs” in this literature review [6]. Most reviews follow this convention, as these articles do not necessarily deduce a specifically endosomal or plasma membrane EV source.

### EV biogenesis

**Fig. 2:**
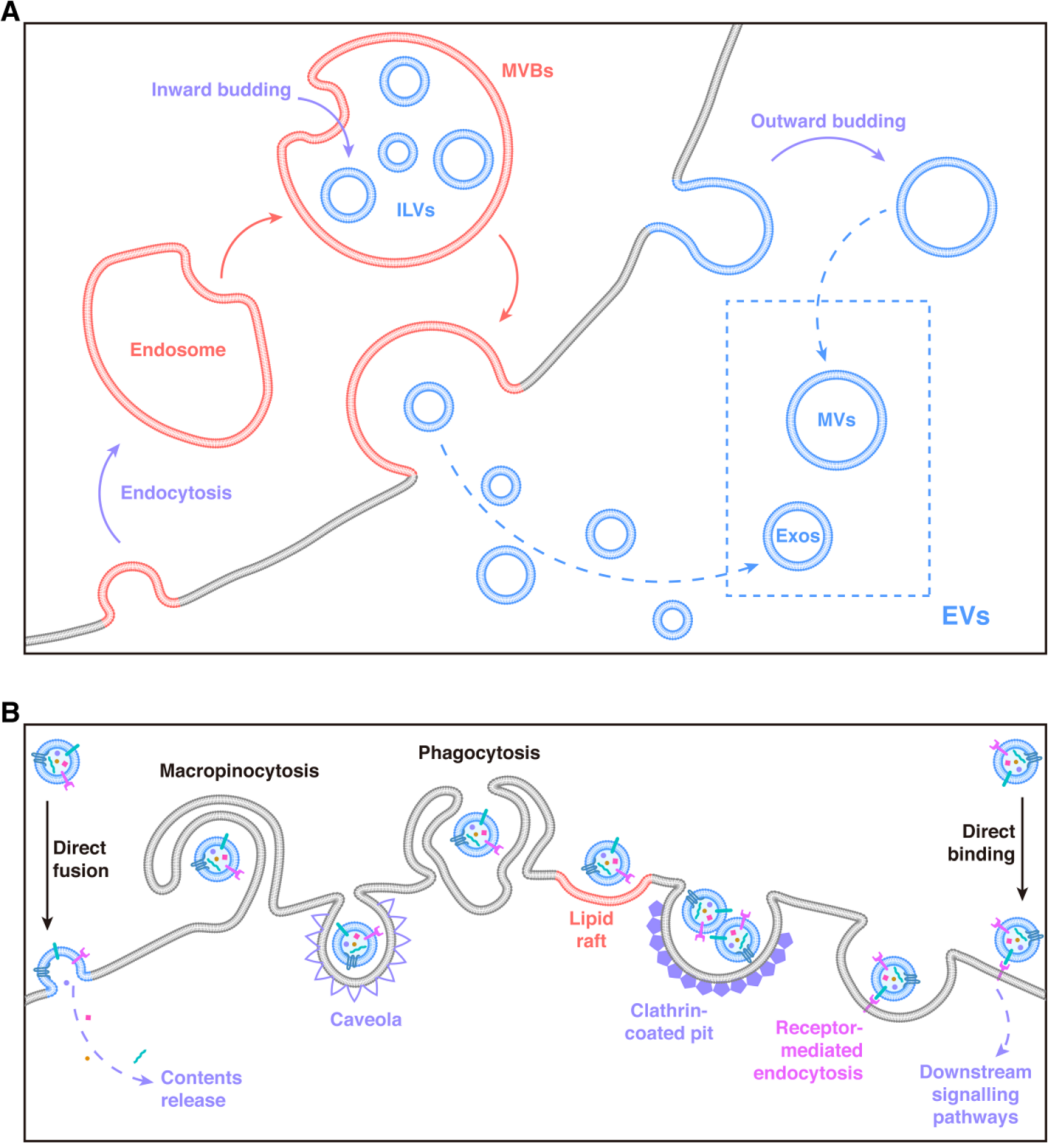
Biogenesis and uptake of extracellular vesicles (EVs). **a** the biogenesis of EVs including exosomes (Exos) and microvesicles (MVs). Exos come into being in MVBs via inward budding, while MVs are directly generated on cytomembrane via outward budding. **b** The uptake mechanism of EVs. The effect of EVs on target cells can be achieved by direct fusion (with contents release into the target cells), “swallow” (including phagocytosis, macropinocytosis, and caveola/lipid raft/clathrin/receptor-mediated endocytosis), and direct binding (membrane proteins directly activate signaling pathways of target cells without “swallow” or content release)

Exos biogenesis consists of a sequence of cellular activities (Fig. 2a). The donor/parental cells initially internalise extracellular ligands and materials to develop endosomes. Exos first come into being as intraluminal vesicles (ILVs) inside the lumen of such endosomes via “inward budding” of the endosomes [33]. After “inward budding” and the selective incorporation of proteins, nucleic acids, and lipids, endosomes are converted to multi-vesicular endosomes (commonly referred to as MVBs) [33, 34]. MVBs are predisposed to fuse with lysosomes for the degradation of their components, thus providing the required materials and energy for cellular activity. Nonetheless, they may additionally fuse with the plasma membrane to release ILVs into the extracellular environment [33]. After release, these ILVs are referred to as Exos.

MV biogenesis differs from that of MVB-derived Exos [33, 35, 36]. In brief (Fig. 2a), MVs are constructed via “outward budding”, division of the plasma membrane, and direct discharge into the extracellular environment [33, 35].

### EVs in the microenvironment

EVs manage many different cellular procedures, such as cell proliferation, survival, and transformation via autocrine and paracrine intercommunication [33, 37]. It is known that EVs work as vehicles for bidirectional intercommunication among cells. The ligands and receptors identified on the surface of EVs offer vector-borne transmission to cells showing the cognate ligand/receptors, providing specificity for this intercommunication [37, 38].

There are several procedures through which EVs and their consignments could be transmitted to recipient cells. EVs can dock at the plasma membrane of a target cell [28, 39], and combined EVs may possibly integrate directly with the plasma membrane of the recipient cell [28, 39]. Furthermore, combined EVs can be picked up by processes such as phagocytosis, macropinocytosis, lipid raft-mediated endocytosis, clathrin-mediated endocytosis, and caveolin-mediated endocytosis (Fig. 2b) [9, 28, 39]. The moment they are endocytosed, EVs can be targeted to lysosomes for degradation [28, 39]. EVs can also integrate with the delimiting membrane of an endocytic compartment, thus permitting the discharge of EV contents into the cytosol of the recipient cells [28, 39]. EVs transport bioactive molecular compounds, which may influence the features and phenotypes of recipient cells by affecting gene expression via de novo translation, post-translational modification of target mRNAs [33, 37], or triggering multiple signalling pathways [37, 39].

Typical functions of EVs include promoting development and growth [40, 41] and the immune avoidance of the embryo in pregnant females [42–44]. EV-mediated bidirectional correspondence between the embryo and uterine endometrium is essential for successful embryo implantation [45], and EVs may control angiogenesis, tissue remodelling, and growth of the placenta [46, 47].

The fast growth of the field of EV study has illuminated unfamiliar mechanisms involving the innate intercellular correspondence systems occurring during malignant tumour commencement and progression [48, 49]. Nevertheless, tumour cells take advantage of these functions of EVs via transformation from a normal microenvironment to the TME. Vesicles which used to support and protect normal tissues then support the growth of tumour tissue, provide nutrient support, and help tumour cells escape the immune system. T-EVs and nm-EVs have been linked in a variety of steps of tumour development (proliferation, angiogenesis, drug resistance, immune escape, and metastasis) [8, 49–52].

## Tumour-derived EVs

### T-EVs to tumour cells

**Fig. 3:**
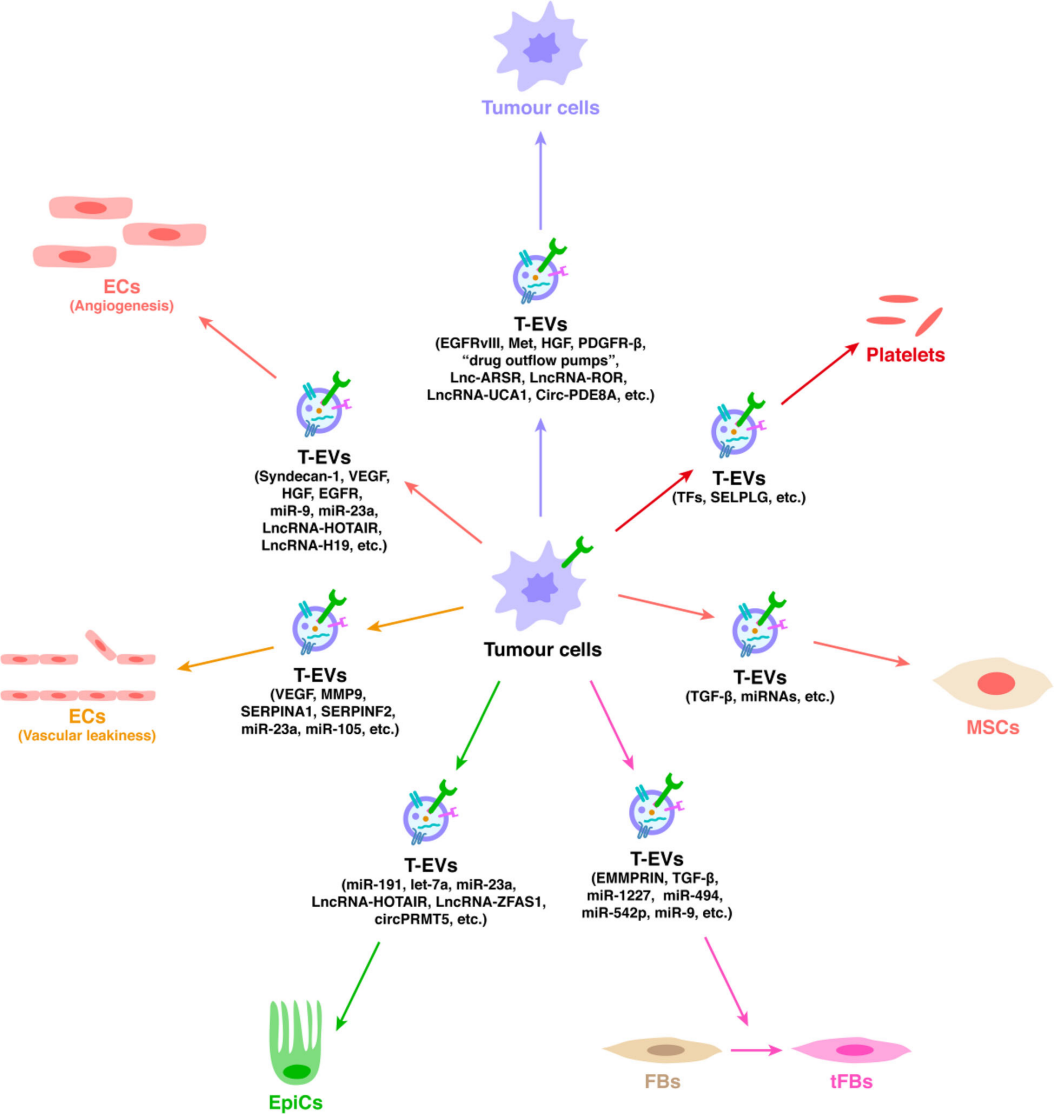
Tumour-derived extracellular vesicles (T-EVs) and their effects on stromal cells. The T-EVs has a variety of effector molecules (including proteins, miRNAs, LncRNAs, and circRNAs), and these molecules are involved in regulation of stromal cells by tumour cells. In addition, there is communication between tumour cells mediated by T-EVs, which many studies have been shown to be associated with cancer resistance and recurrence

T-EVs are able to transmit oncogenic molecules between tumour cells (Fig. 3). Glioma cells expressing epidermal growth factor receptor variant III (EGFRvIII) produce T-EVs carrying EGFRvIII in order to transfer it to EGFRvIII-negative tumour cells inside the same primary tumour [53]. Following T-EV-mediated uptake by recipient cells/tissues, EGFRvIII triggers the mitogen-activated protein kinase (MAPK) and protein kinase B (PKB/Akt) signalling pathways, causing morphological change and boosting malignant tumour development [53]. Subpopulations expressing high levels of Met (Met-high) in melanoma cells show a varied phenotype, resistance to BRAF inhibitors, and increased lung metastasis [54]. T-EV-secreted Met originates from Met-high tumour cells, and augmented Met expression in Met-low tumour cells supports their metastatic capacity in the lungs.

In hepatocellular carcinoma (HCC) invasive cell lines, in vitro and in vivo resistance to Sorafenib is caused by the distribution of hepatocyte growth factor (HGF), with the assistance of T-EVs and the subsequent activation of the HGF/c-MET/PI3K/AKT signalling pathway [55]. Additionally, platelet-derived growth factor receptor-beta (PDGFR-β), which is greatly increased in T-EVs discharged by melanoma cells resistant to the BRAF inhibitor PLX4720, can be transported to recipient melanoma cells. This leads to dose-dependent activation of PI3K/AKT signalling and evasion of BRAF inhibition [56].

Malignant tumour cells are able to transfer resistance via horizontal transmission of T-EVs containing “drug outflow pumps” [57]. Amongst the most thoroughly studied “drug outflow pumps”, T-EVs transporting P-glycoprotein (ABCB1, P-gp, or MDR-1) have been implicated in the transmission of multi-drug resistance to sensitive cellules [58–61]. T-EV-mediated intercellular transmission of effective MRP1 “drug outflow pumps” (ABCC1) has been demonstrated in leukaemia cells [62]. Other “drug outflow pumps” such as ABCG2 or ABCA3 have been shown to be transmitted via T-EVs and to regulate drug resistance in recipient cells [63, 64].

The existence of selective P-gp/MDR-1 mRNA in T-EVs discharged from doxorubicin-resistant osteosarcoma cells suggests that resistant tumour cells employ methods for spreading drug resistance to sensitive cells. This may occur via delivery of MDR proteins directly to sensitive cells or by delivering the mRNA which encodes them [61].

LncRNA also plays an important role in this process. Lnc-ARSR is strongly expressed in sunitinib-resistant renal cell cancer (RCC) cells compared to sunitinib-sensitive RCC cells. EV-carrying Lnc-ARSR competitively binds miR-34 and miR-449, triggering the improved expression of AXL/c-MET and re-activation of STAT3, AKT, and ERK signalling. Triggered AKT causes the transcriptional de-repression of Lnc-ARSR via destruction of FOXO1/FOXO3a, developing a positive feedback loop [65]. T-EVs carrying LncRNA-ROR assist recipient cells in obtaining chemoresistance in HCC by activating the TGF-β signalling pathway. In estrogen receptor (ER)-positive breast cancer cells, T-EVs carrying LncRNA-UCA1 cause tamoxifen resistance [66].

Circ-PDE8A was found to be a highly-expressed circRNA in pancreatic ductal adenocarcinoma (PDAC) [67–70]. Circ-PDE8A easily binds to miR-338, and moderates the pathological function of PDAC via the miR-338/MACC1/MET pathway [70]. Further, scientists have verified that circ-PDE8A may improve tumour invasion via EV-mediated intercommunication, including duodenal invasion, vascular invasion, and liver metastasis [70].

### T-EVs to endothelial cells

#### Angiogenesis

Angiogenesis is the formation of new blood vessels from pre-existing vessels under particular physiological circumstances, including development or in response to tissue damage [71]. In healthy tissues, angiogenesis is firmly controlled by an accurate equilibrium between inhibitory and stimulatory angiogenic signals regulating EC proliferation and migration [71, 72]. An inequality in this particular regulatory network can result in a number of disorders, including malignant tumours [71–73]. T-EVs play a very important role in tumour-related angiogenesis (Fig. 3).

The upregulation of heparanase in myeloma and breast cancer cells is connected with enhanced release of syndecan-1, vascular endothelial growth factor (VEGF), and HGF in T-EVs. This results in increased endothelial infiltration via the extracellular matrix (ECM), and hence enhanced angiogenic activity [74]. T-EVs generated by human lung or colorectal cancer cells transmit oncogenic EGFR to cultured ECs, through which they trigger EGFR-dependent reactions. This leads to activation of the MAPK and AKT signalling pathways, autocrine production, and VEGF signalling, ultimately enhancing angiogenesis [75].

T-EVs regulate angiogenesis in tumours through the release of non-coding RNAs. For instance, T-EVs transporting miR-9 stimulate EC migration and tumour angiogenesis via the reduction of suppressor of cytokine signalling 5 (SOCS5) levels and activation of the JAK/STAT pathway [76]. Furthermore, miR-23a transport causes angiogenesis through SIRT1 targeting in recipient ECs [77]. T-EVs carrying LncRNAs promote the pro-angiogenic ability of circulating angiogenic cells by increasing the expression of both membrane layer molecules and soluble factors [78]. The LncRNA-HOTAIR is highly expressed in glioma cells, and is contained in T-EVs and subsequently transmitted to ECs. LncRNA-HOTAIR then induces angiogenesis by upregulating VEGF-A expression [79, 80]. LncRNA-H19 has been very closely associated with hepatocarcinogenesis [81], hepatic metastases [82], and angiogenesis [83]. T-EVs carrying LncRNA-H19 are actually transmitted to and internalised by ECs, enhancing the angiogenic phenotype and cell-to-cell adhesion by upregulating VEGF production [84].

#### Vascular leakiness

Vascular leakiness is a characteristic of PMN formation [23, 85]. T-EVs seem to play an important role in this process (Fig. 3). Melanoma-secreted T-EVs induce vascular leakiness, inflammation, and recruitment of bone marrow progenitor cells via upregulation of S100a8, S100a9, and tumour necrosis factor α (TNF-α) [86]. Human breast cancer-derived T-EVs increase vascular leakiness in the lungs by upregulating a subset of S100 proteins and triggering Src kinase signalling [87]. T-EVs produced by glioblastoma cells contain high levels of VEGF-A and stimulate EC permeability, vascular leakiness, and angiogenesis in vitro [88]. Proteomics analysis of T-EVs has demonstrated that T-EVs discharge several proteins, including MMP9, SERPINA1, and SERPINF2. The upregulation of these proteins has a substantial role in ECM remodelling, vascular leakiness, and invasiveness [89]. T-EVs originating from lung cancer or breast cancer cells specifically carry miR-23a and miR-105, which both target the tight junction protein ZO-1. This enhances vascular leakiness and the trans-endothelial migration of malignant tumours [90, 91].

More research is required to accurately identify the mechanism by which T-EVs influence the stability of the endothelial barrier, as well as the specificity of this particular targeting within the vasculature of various body organs.

### T-EVs to fibroblasts

Tumour-associated FBs (tFBs; also known as cancer-associated fibroblasts (CAFs)) comprise a large part of the responsive tumour stroma, and carry out essential functions in tumour development. These cells are reprogrammed stromal cells that play a role in malignant tumour initiation, ECM remodelling and advancement, PMN development, and metastasis [92, 93]. In fact, there is evidence to suggest that T-EVs have a close relationship with tFBs (Fig. 3).

T-EVs deliver EMMPRIN to FBs, causing the production of MMPs and allowing tumour invasion and metastasis [47]. T-EVs containing transforming growth factor beta (TGF-β) transform FBs into myofibroblasts (MFBs), triggering vascularisation, tumour growth, and regional invasion [94, 95].

T-EVs, but not those released by non-malignant cells (normal cells), contain crucial enzymes associated with miRNA biogenesis. These enable cell-independent miRNA maturation inside EVs [96]. Inhibition of target mRNA expression (such as PTEN and HOXD10) by transmitted mature miRNAs triggers tumour progression in initially non-malignant cells [96]. Large T-EVs produced by amoeboid tumour cells from RWPE-2 prostate cancer cells are enriched in miR-1227 and can enhance FB migration [97].

Compared to normal FBs, ovarian tumour-adjacent tFBs constantly downregulate miR-31 and miR-214 while upregulating miR-155. Transfecting miRNA mimics (miR-31 and miR-214 mimics) and miRNA inhibitors (miR-155 inhibitors) induces a functional shift of normal FBs into tFBs, while the reverse transfection causes the opposite result, the reversion of tFBs into normal FBs [98]. Successive studies have pointed out that T-EVs alone can lead to the functional and phenotypic changes associated with the conversion of normal stromal FBs into pathogenic tFBs [99]. Transmission of T-EVs carrying miR-494 and miR-542p to lymph node stromal cells and lung FBs resulted in cadherin-17 (Cdh17) downregulation and matrix metalloproteinase upregulation (MMP2, MMP14, and MMP3) [100]. Transmission of the pro-metastatic miR-9 in breast cancer-derived T-EVs bolstered the transformation of human breast FBs to tFBs, leading to strengthened cell motility [101].

### T-EVs to mesenchymal stromal cells

T-EVs are able to stimulate MSCs to differentiate into tumour-supportive cells (Fig. 3) by delivering growth factors, including TGF-β and various miRNAs [19, 102]. Breast cancer-derived T-EVs nurture a myofibroblastic phenotype in adipose tissue-derived MSCs (Ad-MSCs), accompanied by enhanced VEGF, TGF-β, stromal cell-derived factor 1 (SDF-1), and C-C motif chemokine ligand 5 (CCL5) expression [103]. Furthermore, colorectal cancer-derived EVs stimulate tumour-like behaviour in MSCs, which may favour tumour growth and invasiveness [104]. Similarly, EVs originating from osteosarcoma cells carry a high level of TGF-β1, which causes MSCs to secrete interleukin-6 (IL-6). This is connected with enhanced metastatic spread [105].

### T-EVs to epithelial cells

In numerous cell types, epithelial–mesenchymal transformation (EMT) pertains to tumour intrusion and metastasis [106, 107]. Epithelial cells (EpiCs) undergo structural changes after EMT, wherein their polarity is lost. EMT is identified by the acquisition of a mesenchymal phenotype as a result of reduced keratin filaments and reduced E-cadherin expression, as well as increased expression of vimentin, fibronectin, N-cadherin, α-SMA, and various proteases [108, 109]. EMT facilitates tumour cell invasion and migration, making it possible for tumour cells to avert apoptosis.

T-EVs also take part in EMT (Fig. 3). EVs isolated from the metastatic breast cancer cell line MDA-MB-231 promoted linoleic acid stimulation in an EMT-like fashion in MCF10A EpiCs [110]. The function of EVs in regard to cell polarity and EMT initiation in vivo needs to be further investigated [111].

Two miRNAs (miR-191 and let-7a) have been shown to contribute to melanoma cell-derived T-EV-mediated EMT [112]. A collection of miRNAs (specifically miR-23a) are integrated into EMT-associated EVs, and are substantially enhanced in TGF-β-treated mesenchymal lung adenocarcinoma cells [113].

Primary urothelial bladder cancer (UBC) cells were determined to affect the expression of EMT genes by means of EV-carrying LncRNA-HOTAIR. These include *SNAI1*, *TWIST1*, *ZEB1*, *ZO1*, *MMP1*, *LAMB3*, and *LAMC2*. Utilising shHOTAIR in a pair of human bladder cancer cell lines showed that expression of the master regulator of EMT (SNAI1) was dramatically decreased [114]. LncRNA-ZFAS1 expression is increased in gastric cancer cells, and higher ZFAS1 has been correlated with lymph node metastasis and with tumour node metastasis (TNM) stages. ZFAS1 is delivered through T-EVs, promoting gastric cancer expansion and migration by supporting the EMT [115].

A recent study determined that circRNA-circPRMT5 was upregulated in serum and urine EVs from UBC patients. Further investigation determined that circPRMT5 supports the UBC cell EMT by serving as a miR-30c “sponge”. As a result, the expression of its own target gene SNAIL1 and E-cadherin is enriched, allowing the cells to become more invasive [116].

### T-EVs to platelets

It is commonly acknowledged that metastatic growth is associated with the risk of thrombotic issues, which is a major cause of death in malignant tumour patients [117]. Coagulation and platelet accumulation at malignant tumour sites protect against recognition of malignant tumour cells by the immune system, ensuring malignant tumour cell migration and dissemination [118].

EVs associated with coagulation can originate from platelets, inflammatory cells, and malignant tumour cells [119]. Raised circulating levels of EVs containing tissue factors (TFs) and various other coagulation-promoting factors are monitored in malignant tumour patients, and are associated with raised risk of thrombosis [119–121].

Fascinatingly, mutated KRAS and TP53 are associated with raised levels of TFs in T-EVs secreted by human colorectal tumour cells [122]. Pancreatic cancer cell-derived T-EVs containing active TFs and P-selectin glycoprotein ligand 1 (SELPLG) have been revealed to accumulate at locations of impairment, reducing haemorrhage upon injection into living mice [123]. Taken together, these data suggest that T-EVs possess potential pro-thrombotic qualities (Fig. 3) [123] and sustain coagulation activity in malignant tumour development and metastasis [124].

### T-EVs to immune cells

**Fig. 4:**
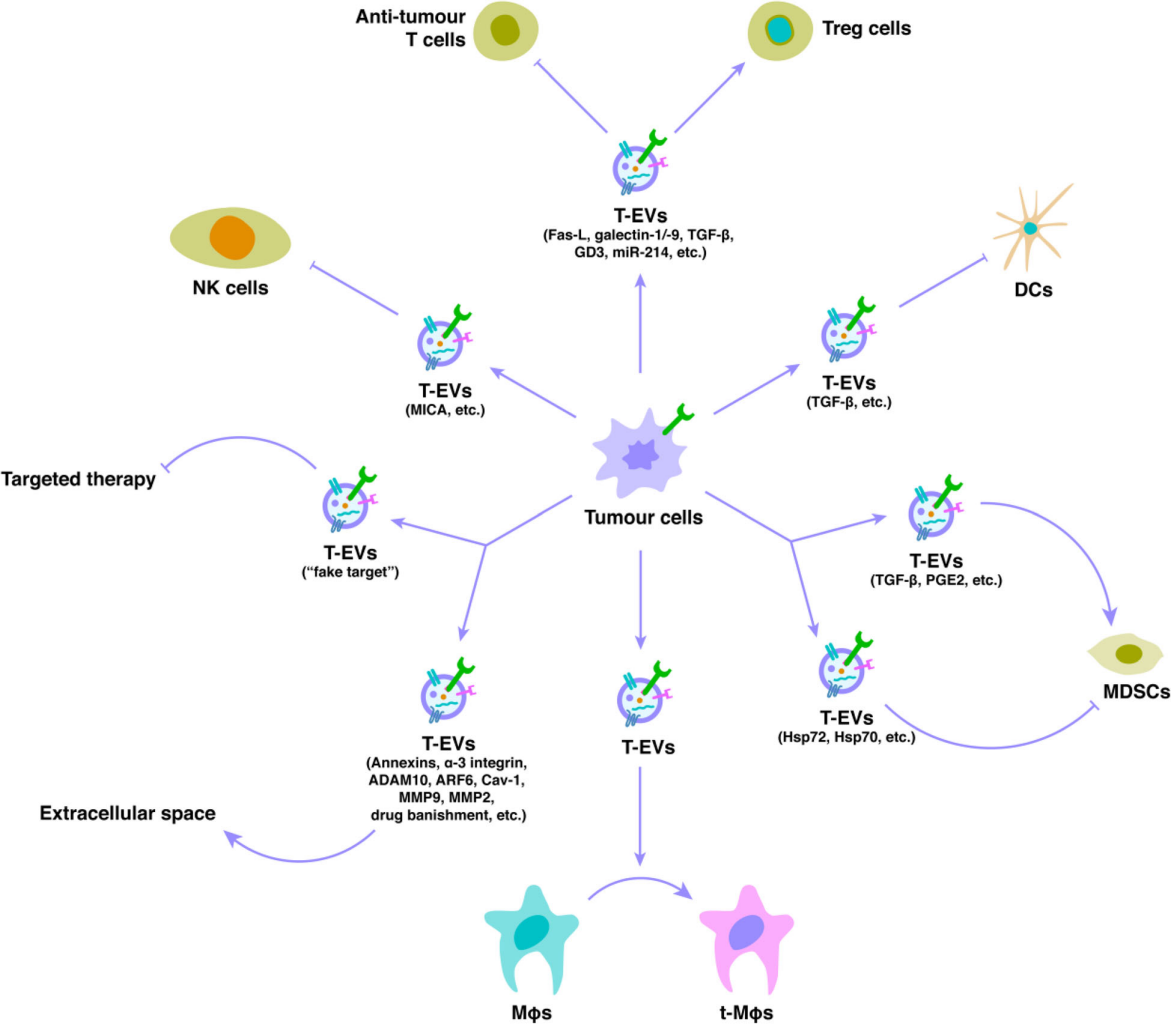
Tumour-derived extracellular vesicles (T-EVs) and their effects on immune cells. T-EVs through the delivery of their content to regulate the function of immune cells (including inhibition of T cells, inhibition of NK cells, activation of Treg cells, inhibition of DCs, bidirectional regulation of MDSCs, and regulation of Mφs polarization). In addition, tumour cells could also use T-EVs as “fake target” to cheat the targeted therapy, use T-EVs to release factors and proteins to affect the extracellular space, or use T-EVs for the drug banishment to evade the attack of anti-tumour drugs

The TME is penetrated by a range of immune cells, including lymphocytes (T cells, B cells, natural killer (NK) cells, and T regulatory (Treg) cells), dendritic cells (DCs), monocytes, Mφs, myeloid-derived suppressor cells (MDSCs), and granulocytes (neutrophils, basophils, eosinophils, and mast cells). The major function of these cells is to ensure immune supervision. Nevertheless, tumour cells are efficient in regulating signalling pathways within these immune cells, turning them into an immunosuppressive entity and resulting in improved malignant tumour cell survival and proliferation [125]. There is a growing body of evidence suggesting the importance of T-EVs in tumour-associated abnormal immunity (Fig. 4).

One study determined that T-EVs induce immunosuppression by promoting apoptosis of hematopoietic stem cells (HSCs), DCs, and peripheral blood lymphocytes (PBLCs) [126]. Many T-EVs have been shown to be enriched for Fas ligand (Fas-L), which causes apoptosis when it binds to its receptor. Fas-L(+) T-EVs cause immunosuppression by enhancing Treg cell expansion and anti-tumour T cell apoptosis, resulting in immune escape [127–131]. The existence of various other mediators of T cell apoptosis in T-EVs, such as galectin-1/− 9, has been shown to trigger T cell apoptosis and immunosuppression [132, 133].

T-EVs carry TGF-β externally and transport it to T cells, suppressing their proliferation in response to IL-2 and altering their phenotype to Treg cells [134, 135]. Furthermore, T-EVs inhibit the differentiation of monocytes into DCs and enhance the production of a TGF-β-producing myeloid immunosuppressive cell subset—MDSC—which then suppresses T lymphocyte proliferation [136]. The enrichment of prostaglandin E2 (PGE2) and TGF-β in T-EVs causes the accumulation of MDSCs with immune suppressive features [137]. It has also been revealed that T-EV-associated Hsp72 or Hsp70 mediate inhibition of MDSCs through STAT3 activation [138, 139].

These T-EVs have been revealed to trigger DCs and cause IL-6 secretion, which enhances tumour invasion by increasing MMP-9 metalloproteinase expression [140]. T-EVs are able to induce IL-6 production inside monocytes via toll-like receptor (TLR) activation. IL-6 then triggers the signal transducer and activator of transcription 3 (STAT3) pathway in immune cells, stromal cells, and tumour cells. This sustains the general immune escape of malignant tumour cells [141]. Likewise, tumour cells are able to discharge T-EVs containing MHC class 1-related chain ligand A (MICA). This can bind to the NK cell receptor NKG2D, resulting in its downregulation and leading to a significant decrease in NK cytotoxicity, independent of target cell NKG2D ligand expression [142]. One study recently discovered that GD3, a ganglioside expressed on the surface of T-EVs, arrests T cells by engaging their T cell receptor (TCR) [143].

A previous study confirmed that PD-L1 exists in EVs derived from the urine or blood of patients with early IgA nephropathy [144]. Research has affirmed that the amounts of PD-L1 expressed on EVs, but certainly not dissolvable PD-L1, are associated with the advancement of head and neck squamous cell carcinoma (HNSCC) [145]. Chen et al. have also determined that PD-L1 on metastatic melanoma-derived T-EVs hinders CD8(+) T cell activation and assists with tumour development. This could be interrupted by means of anti-PD-1 monoclonal antibody (mAB) treatment [146]. In HNSCC patients, PD-L1-high EVs considerably inhibited CD69 on CD8(+) T cells [145]. In a prostate cancer syngeneic model, mice were not reactive to anti-PD-L1 mAB therapy as a result of PD-L1-carrying EVs. In 4 T1 tumour models, the accumulation of PD-L1-carrying EVs in the TME caused resistance to immunotherapy by subduing granzyme B secretion. Rab27a knockdown (KD) in tumour cells considerably enriched the performance of anti-PD-1 treatment and inhibited 4 T1 tumour development [147].

Tumour-released miRNAs have similarly been associated with immunosuppression. For example, miR-214 carried by T-EVs was effectively transported into recipient T cells. An in vivo study has shown that miR-214 mediates Treg cell expansion, causing increased immunosuppression and tumour growth in mice [148].

Mφs are multifunctional antigen-presenting cells characteristically classified into a pair of polarised phenotypes: pro-inflammatory (M1) and anti-inflammatory (M2) [149]. Tumour-associated Mφs (t-Mφs) are of the M2 subtype (M2-Mφs) and penetrate malignant tissues [150]. Inside the TME, t-Mφs produce IL-4/5/6, which enhance angiogenesis, matrix remodelling, and immunosuppression [151]. When T-EVs are phagocytosed by undifferentiated Mφs, they undergo M2 polarisation via the suppressor of cytokine signalling (SOCS) 4/5/STAT3 pathway [152]. Pancreatic cancer (PC) cell-derived T-EVs change the differentiation of Mφs to M2-Mφs, ensuring immunosuppression and metastasis occurs independently of HIF-1 and HIF-2. Furthermore, PC-derived T-EVs activate the PI3Kγ pathway to improve immunosuppressive gene expression in M2-Mφs [153].

### T-EVs to extracellular spaces

Throughout the process of malignant tumour progression, the molecular and cellular environments of stromal cells and their extracellular proteins and enzymes are dynamically changing as a result of the influence of T-EVs (Fig. 4). ECM remodelling is commonly believed to enhance the invasive phenotype of tumours. T-EVs carry the ECM compound fibronectin, thus supporting incipient adhesion assembly and boosting cellular motility [154]. Proteomic analysis of T-EVs showed that annexins, α-3 integrin, and ADAM10 were enriched in T-EVs, and were associated with regional invasion and cell migration [155]. Large T-EVs likewise harbour abundant bioactive molecules associated with regional invasion (such as ARF6, Cav-1, MMP9, and MMP2), and their abundance is also associated with tumour development [156].

Research has revealed that EVs participate in invasion and metastasis by means of invadopodium formation [157, 158]. Invadopodia are vibrant actin-rich membrane protrusions which tumour cells generate to invade and degrade the ECM [157]. It was recently suggested that invadopodia are docking sites for EVs, expediting ECM degradation by means of localised secretion of metalloproteinase MT-1-MMP and therefore advancing cell invasion [159, 160]. Similarly, the migration of tumour cells throughout tissues and chemotactic gradients is induced by the formation and release of fibronectin-bound EVs at the leading edge of migrating cells. These fibronectin-bound EVs enhance adhesion assembly and stabilisation, enabling persistent and directional tumour cell migration [154, 161].

EVs could be used as carriers by malignant tumour cells to promote drug resistance via drug sequestration and banishment (Fig. 4). Shedden et al. were the first to mention a positive correlation between the expression of genes related to EV shedding and drug resistance in various malignant tumour cell lines [162]. In a breast cancer cell line, they used light microscopy and flow cytometry to demonstrate that the fluorescent chemotherapeutic agent doxorubicin was physically encapsulated in EVs and ejected into the extracellular medium [162]. More recently, melanoma cells became resistant to cisplatin treatment via an extracellular acidification-mediated increase in EV secretion and the direct export of cisplatin into these EVs [163]. Cisplatin was discovered to be removed from resistant ovarian carcinoma cells via EVs [164]. B-cell lymphoma cells additionally effectively expelled doxorubicin and pixantrone in T-EVs in vitro [165].

Malignant tumour cells can also make use of EVs as “fake targets”, thus weakening targeted treatments (Fig. 4). T-EVs transport a huge selection of cellular antigens, all of which are presented in an orientation identical to those found on the surface of the cells from which they originate. On the surface of EVs, the existence of antigens targeted by immunotherapy acts as a sink for monoclonal antibody-based drugs, thus reducing their bioavailability to their anticipated target.

This is exemplified by B-cell lymphoma, when the existence of CD20 on the surface of EVs protects targeted lymphoma cells from rituximab (an anti-CD20 mAB) [63]. Both in vitro and in vivo research into breast cancer has demonstrated the function of HER2(+) EVs in regulating resistance to the anti-HER2 mAB Trastuzumab. T-EVs produced by either HER2(+) tumour cells in vitro or discovered in the serum of breast cancer patients bind to Trastuzumab, thus impeding its activity in vitro [166].

**Fig. 5:**
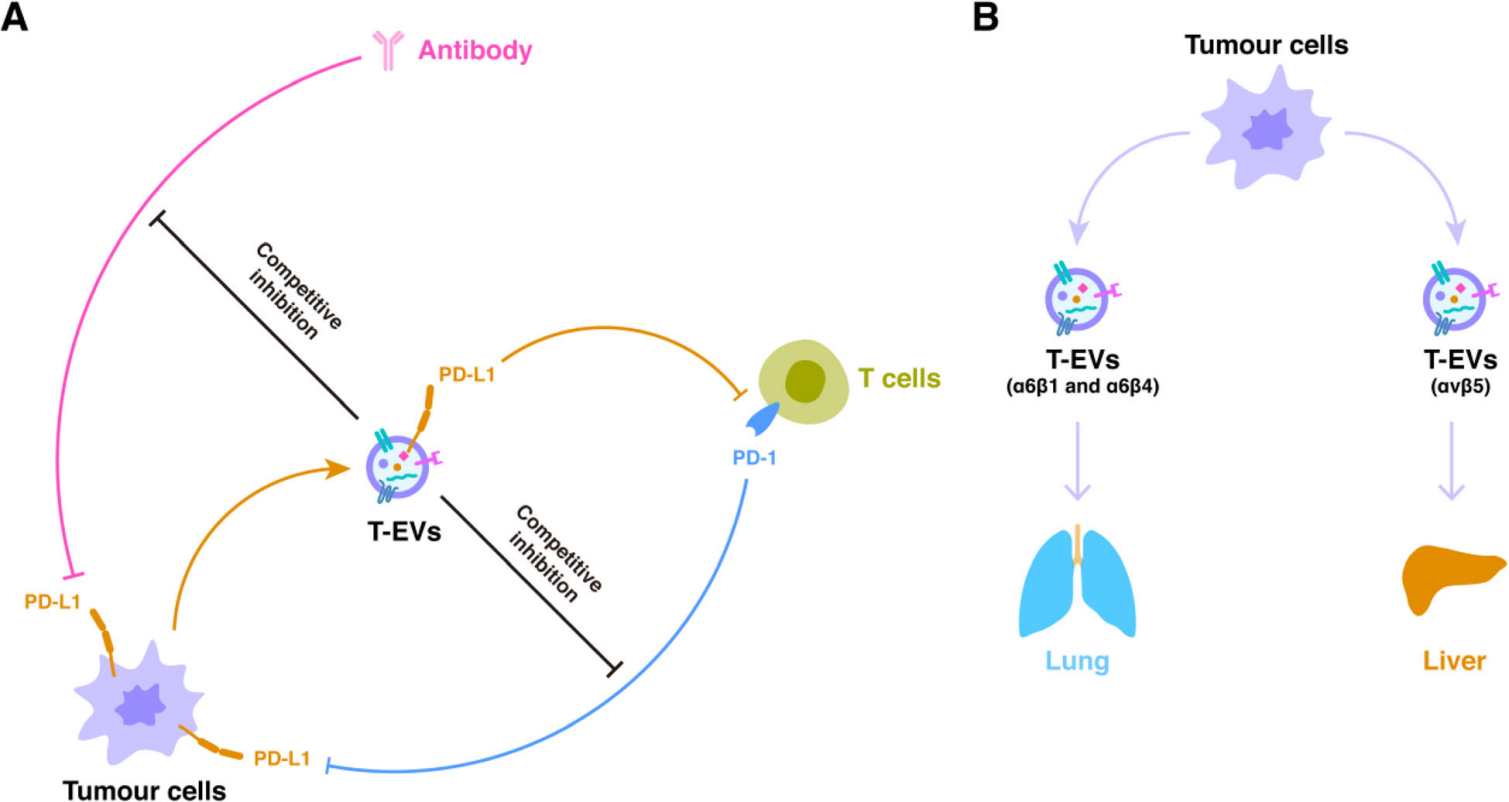
Tumour-derived extracellular vesicles (T-EVs) and their effects related to specific recognition. **a** T-EVs and PD-1/PD-L1 network. T-EVs can carry PD-L1 on their surface to competitively combined PD-1 on T cells, to suppress the attacks from T cells on tumour cells. In addition, PD-L1-carrying T-EVs could also play a “fake target” to neutralize the therapeutic effect of PD-L1-targeting antibody drugs. **b** T-EVs and their organ targeting ability. For example, α6β1/α6β4-carrying T-EVs are targeting to lung, while αvβ5-carrying T-EVs are targeting to liver

Immune checkpoint blockade therapies feature anti-CTLA-4 monoclonal antibody (mAb), anti-PD-1 mAb, and anti-PD-L1 mAb [167]. It is largely recognised that a PD-1/PD-L1 blockade could possibly trigger T cells. However, little has been discovered about the role of PD-L1-carrying EVs in the relatively low response rate to anti-PD-L1/PD-1 treatment [168]. The interruption of intercommunication between the checkpoint ligand (such as PD-L1) and the inhibitory checkpoint receptor (PD-1) on T cells restores T cell function and anti-tumour immunity. Nevertheless, not all patients respond to this type of immune checkpoint inhibitor treatment. The presence of the checkpoint ligand (PD-L1) on T-EVs soon after treatment categorises melanoma patients as either responders or resistant to anti-PD-1 treatment [146]. T-EVs steer this type of antibody far from the tumour by securing the immunotherapeutic antibody on their surface, leaving it free to face PD-1 on approaching tumour-specific T cells. The same machinery has been used to explain glioblastoma in vitro, in which T-EVs exhibit PD-L1 and suppress both T cell proliferation and antigen-specific T cell responses [169]. In a prostate cancer mouse model, mice were not reactive to anti-PD-L1 mAB therapy as a result of EVs carrying PD-L1. In 4 T1 tumour models, the accumulation of PD-L1 on EVs in the TME caused immunotherapy resistance by subduing granzyme B secretion. Significantly, Rab27a KD in tumour cells considerably improved the performance of anti-PD-1 treatment and inhibited 4 T1 tumour development [147]. In HNSCC patients, PD-L1-high EVs considerably hinder CD69 on CD8(+) T cells, which may also be obstructed by anti-PD-1 antibodies [145]. In Fig. 5a, we present a diagram summarising and demonstrating the role of T-EVs in interference of regular PD-1/PD-L1 interactions.

### PMN build-up

Metastasis is a multi-step procedure resulting in the spread of primary tumour cells to distant body organs. T-EVs have been associated with all steps of tumour invasion and metastasis [159, 170–172]. PMNs accumulate by the means explained in the previous sections and possess some characteristic functions.

T-EVs possess their own protein “postal code” of specific integrin profiles (Fig. 5b). This directs them to specific body organs, thereby deciding their metastatic organotropism [87]. The metastatic organotropism and building of a PMN is determined by T-EVs secreting various sets of integrins (including α6β1, α6β4, or αvβ5), which preferentially fuse tumour cells with resident cells at their anticipated location. T-EVs taken up by organ-specific cells prepare the PMNs, and specific integrin patterns predict the organotropism of tumour cells, integrins α6β1 and α6β4 as being related to lung metastasis. However, integrin αvβ5 has been determined to be related to liver metastasis [87].

CircRNAs in blood EVs, called ciRS-133, are closely associated with the light browning of white adipose tissue (WAT) and malignant tumour-associated cachexia. After being provided to pre-adipocytes, ciRS-133 reduce miR-133 expression, activate PRDM16, and promote the differentiation of preadipocytes into brown-like cells. It has been demonstrated that ciRS-133 KD may prevent tumour-implanted mice from struggling with malignant tumour-related cachexia, demonstrating the contribution of EV-circRNAs in tumour pathogenesis [173].

## EVs from non-tumour cells

**Fig. 6:**
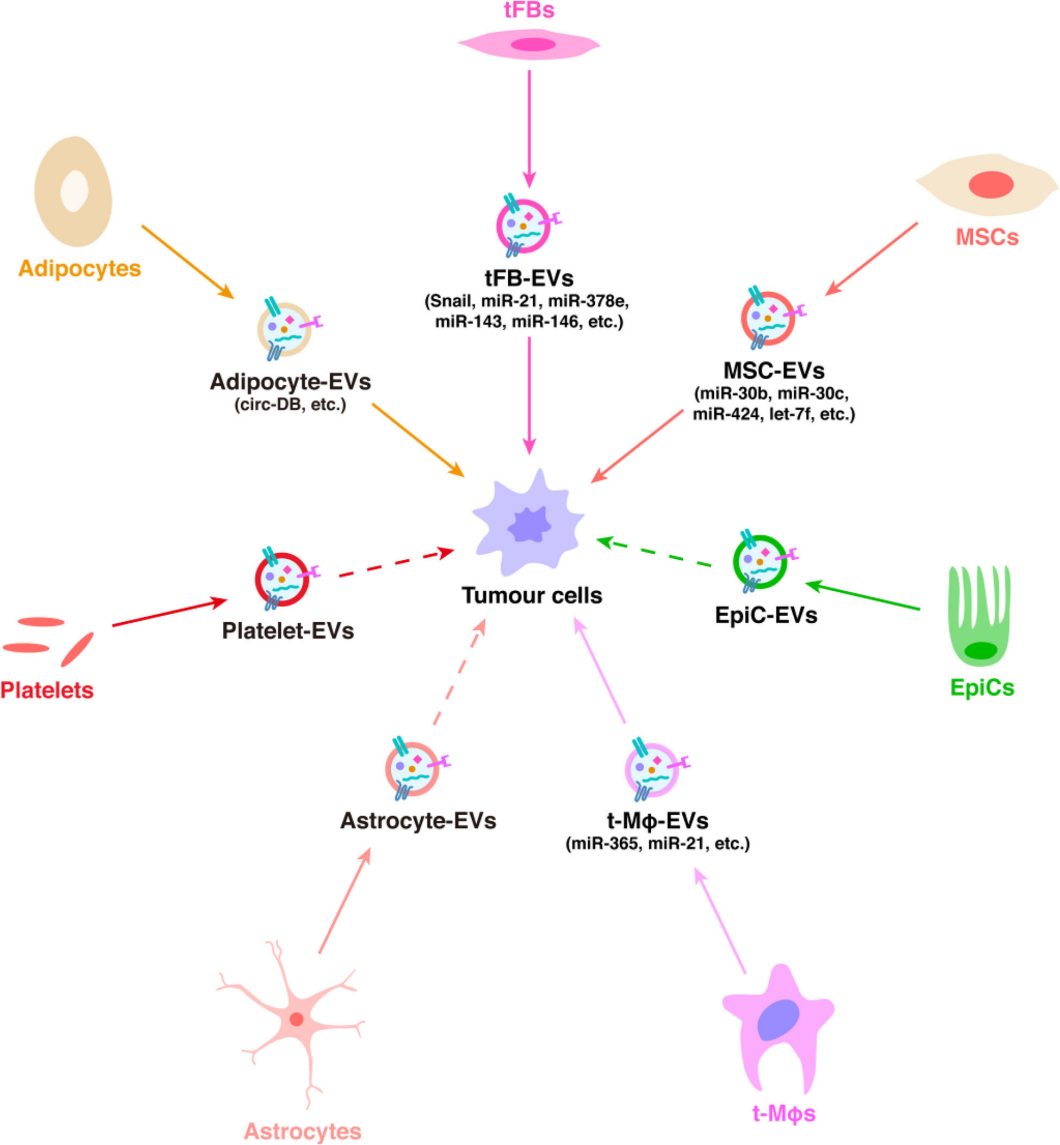
Extracellular vesicles derived from non-malignant cells (nmEVs) and their effects on regulation of malignant tumours’ behaviour. Current research suggests that nmEVs derived from tFBs, adipocyte, platelets, astrocyte, t-Mφs, EpiCs, and MSCs plays a crucial role in tumour progression and metastasis, some important molecules in nmEVs are identified in nmEVs

Tumour expansion and drug resistance are not only decided by malignant tumour cells but are also sustained by non-tumour cells inside the TME. Hence, it is quite reasonable to think that nmEVs also play an important role in affecting the TME (Fig. 6). Thus tFB-derived EVs (tFB-EVs) may reinforce tumour growth, survival, invasion, and metastasis. By producing chemoresistance-inducing EVs enclosing Snail and miR-146, pancreatic tFBs, which are fundamentally resistant to the chemotherapeutic agent gemcitabine, mediate the transmission of resistance to pancreatic cancer when exposed to gemcitabine. This enhances their proliferation and survival [174]. tFB-EVs may also magnify breast cancer protrusive activity, motility, and metastasis by triggering autocrine Wnt-planar cell polarity (PCP) signalling [175]. Studies have determined that three miRNAs (miR-21, −378e, and also − 143) are upregulated in tFB-EVs and can be easily transferred into breast cancer cells to promote EMT [176]. Similarly, the transposition of miR-21 from tFBs to ovarian cancer cells minimises apoptosis and elevates paclitaxel chemoresistance by downregulating apoptotic peptidase activating factor (APAF1) mRNA expression [177].

MSC-derived EVs (MSC-EVs) may generate drug resistance in gastric cancer cells by activating the CaM-Ks/Raf/MEK/ERK signalling pathway [178]. EVs carrying RNA from stromal cells, which are mainly transposable elements and non-coding transcripts, may be transported to breast cancer cells. This leads to an increase in therapy- and radiation-resistant breast cancer cells via a mechanism requiring NOTCH3 induction [179]. MSC-EVs with pro-angiogenesis miRNAs (miR-30b, 30c, 424, and let-7f) can easily upregulate the expression of pro-angiogenic factors in cancer cells [180].

HRAS overexpression in EpiCs boosts the packing of mesenchymal markers (including vimentin and MMPs) in EVs, possibly causing EMT in recipient cells [181].

Transfer of miR-365 in Mφ-derived EVs (Mφ-EVs) causes pancreatic adenocarcinoma cells to become resistant to gemcitabine in vitro and in vivo [182]. M2-Mφs (t- Mφs)-derived miR-21 secretion confers cisplatin resistance in gastric cancer cells. Functional investigations have disclosed that EVs carrying miR-21 may be transported directly from Mφs to gastric cancer cells, where they inhibit programmed cell death and increase PI3K/AKT signalling pathway activation via PTEN downregulation [183].

The transfer of miRNAs specifically targeting PTEN expression from astrocyte-derived EVs to invading tumour cells in the brain microenvironment supports brain metastasis, despite the fact that other autocrine and paracrine signalling may also be coordinated throughout tumour development [184].

CircRNAs are commonly expressed in individual cells such as blood cells [185, 186]. Researchers extracted EVs from platelets and determined that circRNAs are selectively packaged and discharged directly into EVs. Given that platelets participate in different physiological procedures including neoplasm, inflammation, and coagulation metastasis, EV-circRNAs could be transported throughout the body to participate in a variety of regulatory functions [187]. It was also noted that some EV-circRNAs derived from adipose cells can easily influence de-ubiquitination in HCC. Still more EV-circ-de-ubiquitination (circ-DB) occurs in patients with greater rates of body fatty tissue. Research has shown that circ-DB switches on USP7 in HCC cells by lessening the degree of miR-34a expression. The circ-DB/miR-34a/USP7/CyclinA2 signalling pathway was discovered by this means, through which the EV-circRNAs upregulated malignant tumour development and inhibited DNA damage [188].

## EVs and diagnosis

Given that they are incredibly stable, abundant, and tumour-specific, T-EVs have numerous unique advantages as biomarkers [189]. One interesting T-EV biomarker is the epithelial cell adhesion molecule (EpCAM) [190]. EpCAM(+) T-EVs enhance malignant ovarian tumour development, and are significantly more numerous in patients with malignant ovarian tumours than in females with benign ovarian disorder or healthy control subjects [191].

EV integrins (as a counterpart to tumour-expressed integrins) can act as biomarkers to predict the probability of malignant tumours, in addition to determining metastatic tendencies in specific organ sites [87]. Specific EV integrin mixtures determine organ-specific metastasis. The α6β4 and α6β1 EV integrins are associated with lung metastasis, αvβ5 EV integrins with liver metastasis, and αvβ3 EV integrins with brain metastasis models [87].

Circulating T-EVs from patients with stage IV melanoma carry a protein signature composed of the melanoma-specific protein tyrosinase-related protein-2 (TYRP2), very late antigen 4 (VLA-4), HSP70, and MET oncoprotein [86]. T-EVs from the plasma of melanoma patients are enriched in caveolin-1 compared with healthy controls, suggesting that caveolin-1(+) T-EVs are likely to be another prospective melanoma biomarker [192]. EV glypican-1 has likewise been suggested as a prognostic and diagnostic indicator for pancreatic malignant tumours [193]. In patients with pancreatic ductal adenocarcinoma (PDAC), the amount of the protein MIF inside T-EVs may represent a prognostic marker for liver metastasis. Circulating T-EVs from stage-I PDAC patients that later showed established liver metastasis had been improved by higher levels of MIF, as compared with patients whose cancer did not advance and healthy control cases [194].

Research has illustrated that the detection of PD-L1(+) EVs in serum is correlated with poor prognosis in individuals with pancreatic ductal adenocarcinoma [195]. New findings have suggested that miR-21 contained in PD-L1(+) EVs possesses the potential to become a biomarker for distinguishing between NSCLC patients and healthy controls [196].

Additionally, EVs transport single-stranded DNA (ssDNA), which summarises genomic eccentricities such as oncogene amplifications (such as MYC) in the primary tumour [197]. In the metastatic environment, a higher level of double-stranded DNA (dsDNA) was discovered in T-EVs in aggressive melanoma compared to melanoma with reduced metastatic capacity or in non-metastatic melanoma [198]. T-EV dsDNA reflects the oncogenic mutational condition of the particular parental malignant tumour cell [193, 198, 199]. This emphasises the utility of T-EV dsDNA as a biomarker for diagnosing oncogenic mutations in a clinical setting.

Tumour-specific mRNA isolated from T-EVs from the serum and tissue of glioblastoma patients reflects the mutational condition of EGFRvIII [200, 201]. MiRNAs within circulating T-EVs possess prognostic and/or diagnostic value for many types of malignant tumours. T-EVs carrying miR-373 are primarily enhanced and overall greater in triple-negative breast cancer patients, highlighting the potential role of miR-373 as a plasma-based biomarker for more hostile tumours [202]. Serum EVs carrying numerous miRNAs are considerably higher in individuals with primary malignant tumours compared to normal controls, including miR-21 and miR-125b [203, 204]. In patient serum, EV miR-17-92a cluster expression levels are associated with recurrence of colon cancer, while EV miR-19a is associated with poor prognosis [205]. EV miR-141 and miR-375 have been connected with metastatic prostate cancer [206, 207]. An additional study determined the association between higher levels of miR-1290 and miR-375 in serum EVs and reduced survival in patients with castration-resistant tumours [208]. When comparing EVs from metastatic sporadic melanoma patients to those in familial melanoma patients or unaffected control subjects, miR-17, miR-19a, miR-149, miR-21, and miR-126 were expressed at greater levels in the former [209].

LncRNAs associated with T-EVs are also appealing as prospective biomarkers. Nevertheless, LncRNAs in EVs may work as biomarkers in various other malignant tumours, such as LncRNA-p21 in prostate cancer and LncRNA-HOTAIR in bladder cancer [210]. In colorectal cancer (CRC), LncRNA-MAGEA3 has been determined to be a colorectal cancer-related serological biomarker. EVs carrying LncRNA-CRNDE-h are increased in the serum of CRC individuals, and have been associated with factors connected to poor CRC prognosis [211]. Additionally, high levels of LncRNA-CRNDE-p and reduced miR-217 on serum EVs are associated with enhanced medical stages (III/IV), tumour classification (T3/T4), and lymph node or remote metastasis [212]. Blood LncRNA-LINC00152 is substantially higher in gastric cancer (GC) patients compared to healthy controls [213]. A similar study illustrated that ZFAS1 is highly expressed in the serum EVs of GC patients. ZFAS1 upregulation is also connected with the TNM stage and lymphatic system metastasis. This demonstrated that EVs carrying ZFAS1 may act as a prospective diagnostic biomarker for GC [214].

Nonetheless, the features of circRNAs mark these molecules as a better option for detecting disorders due to their closed conformation and resistance to RNase. Compared to the 48-h half-life of the majority of circRNAs, the ordinary half-life of miRNAs is normally less than 10 h [215]. Scientists extracted circulating T-EVs originating from PDAC patients and determined that higher EV-circPDE8A expression was closely related to duodenal infiltration, vascular infiltration, and the TNM stage [70].

With recent technological improvements, microfluidic technology has been introduced into the field of EV study. Compared with traditional methods, microfluidic technology can carry out EV-based diagnosis more easily, efficiently, and economically [216].

## EVs and therapy

### Elimination of detrimental EVs

EV biogenesis is a major target for EV-targeting therapy in malignant tumour treatment [217–219]. A number of Rab proteins have been revealed to be associated with the selective packing and generation of EVs in both normal cells and tumour cells [28, 119, 220]. Rab27a KD in metastatic melanoma and malignant breast tumour cells resulted in a significant decline in EV generation, primary tumour sizing, and metastasis [86, 218]. Therefore, identifying the profile of Rab proteins responsible for EV release in malignant tumour cells could result in novel therapeutic options.

Federici et al. carried out therapy with a proton pump inhibitor to observe the effects of both cisplatin uptake and EV release in vitro and in vivo*.* In a mouse xenograft model of melanoma, they demonstrated that therapy with a proton pump inhibitor reduces the release of EVs and enhances tumour cell sensitivity to cisplatin [163]. Numerous inhibitors of EV release, such as a calpain inhibitor [221], prevent EV release in response to calcium mobilisation. This was observed in prostate cancer cell lines in vitro, and enhanced sensitivity of cells to chemotherapy was observed in vivo [222]. Inhibition of EV release by avoiding the activation of ERK via a MEK inhibitor led to enhanced sensitivity of pancreatic cancer cell lines to gemcitabine in vitro, and in a tumour graft model in vivo [223].

While many of the agents specifically blocking T-EV release from malignant tumours lack specificity, some inhibitors target tumour-specific enzyme isoforms. This is the case for peptidylarginine deiminase (PAD)2 and PAD4 inhibitors, which are overexpressed in prostate and ovarian malignant tumour cells. Their inhibition by chloramidine minimises T-EV production, thus increasing the sensitivity of malignant tumour cells to chemotherapy drugs [224]. In a more methodical in vitro study, Kosgodage et al. disturbed T-EV biogenesis in prostate and breast cancer cell lines. They determined that amongst a collection of 11 inhibitors targeting different steps of T-EV biogenesis, PAD inhibitors and PKC (bisindolylmaleimide-I) inhibitors were the most effective [225]. The same group recently demonstrated the impressive role of cannabinol (CBD) as an inhibitor of T-EV release in prostate, hepatocellular carcinoma, and breast cancer cell lines. The CBD-induced inhibition of T-EVs significantly escalated cell sensitivity to anti-cancer drugs including doxorubicin and pixantrone [226].

Although these treatments have had success in vitro and sometimes in vivo, their lack of selectivity for malignant tumour cells restricts their therapeutic usage. This is not the case for the specific elimination of circulating T-EVs from plasma.

In a technique quite similar to haemodialysis, extracorporeal hemofiltration with cartridges composed of hollow fibres (with a size cut-off of 200 nm) combined with an affinity matrix allows specific elimination of ultra-filtered EVs. This procedure is known as Adaptive Dialysis-like Affinity Platform Technology (ADAPT™), and was first developed by Aethlon Medical Inc. for eliminating Hepatitis C virus (HCV) particles from the bloodstream of contaminated patients [227]. The expansion of this approach to the specific elimination of EVs with a hollow fibre size cut-off lower than 200 nm, has been discussed by Marleau and colleagues [228].

### Use of EVs

Activation of anti-tumour T cell reactions by DC-derived EVs (DC-EVs) has been determined to be critical in reducing the expansion of well-established tumours [229]. Loading DC-EVs with MHC/tumour antigen has been carried out for phase I clinical trials in patients with advanced melanoma [230] and non-small-cell lung carcinomas [231]. EVs from B lymphoma cells have been confirmed to have high amounts of HSP70 as well as HSP90, therefore enhancing the anti-tumour immune response [217].

EVs may be therapeutically targeted to supply anti-tumour cargos to malignant cells [232]. Based on their combination of surface proteins, EVs can be routed to specific tissues [87, 194]. These characteristics make them efficient nano-vehicles for the biodelivery of therapeutic RNAs, proteins, and other agents.

Capitalising on EVs, researchers have the ability to target medications to tumour cells. EVs may raise the therapeutic index of doxorubicin (DOX). EVs carrying doxorubicin (EV-DOX) avoid cardiac toxicity by partly restricting the crossing of DOX via myocardial ECs [233]. Another study demonstrated that bovine milk may be a scalable resource for EVs that can easily function as transporters for chemotherapeutic/chemopreventive agents. Comparing the use of soluble drugs, drug-loaded EVs had considerably greater efficiency compared to lung tumour xenografts in vivo [234].

An in vivo study revealed that neuron-targeted EVs packed with Bace1 siRNAs specifically and significantly decreased Bace1 mRNA (60%) and protein (62%) in nerve cells [235]. Similarly, EVs loaded with artificial siRNA targeting MAPK could efficiently knock down the MAPK1 gene at the time of their transmission into lymphocytes and monocytes in vitro [236]. The level of RAD51 transcript significantly decreased in HEK293 and HCT116 colon cancer cell lines when incubated with EVs transporting siRNA targeting RAD51 by electroporation [237]. EVs with si-HGF-1 substantially reduced HGF and VEGF expression, thereby preventing gastric cancer progression [48].

These results suggest that EVs may indeed be beneficial as drug delivery tools. Although several anti-tumour therapies have been investigated/tested in preclinical models and phase I clinical trials, these studies have reinvigorated the desire for novel anti-cancer therapies.

## Discussion and outlook

In the previous sections, we introduced the role of EVs in the TME, including the role of T-EVs and EVs from non-malignant cells. Some representative contents of EVs, their functions and mechanism are shown in Table 1. We also discussed the application of EVs in the diagnosis and treatment of various cancers. Increasing amounts of research have been conducted on this topic, and many interesting findings and perspectives have been presented, in addition to the emergence of new diagnostic and therapeutic techniques. However, there are still gaps in our knowledge and questions that must be addressed.

**Table 1:** Some representative contents of EVs, their functions and mechanism

Disease	EV Contents	Pathways/Mechanism	Function
Glioma	EGFRvIII	MAPK, Akt	tumour development
	LncRNA-HOTAIR	upregulating VEGF-A expression	angiogenesis
Glioblastoma	VEGF-A	–	vascular leakiness, angiogenesis
Melanoma	Met	–	resistance lung metastasis
	PDGFR-β	PI3K/AKT	resistance
	–	upregulation of S100a8, S100a9, and TNF-α	vascular leakiness
	miR-191 and let-7a	–	EMT
	PD-L1	competitive inhibition	Anti-PD-1 therapy resistance
HCC	HGF	HGF/c-MET/PI3K/AKT	resistance
	LncRNA-ROR	TGF-β	resistance
Osteosarcoma	P-gp/MDR-1 mRNA	–	resistance
	TGF-β	secreting IL-6	tumour metastasis
RCC	Lnc-ARSR	STAT3, AKT, ERK	resistance
Breast cancer	LncRNA-UCA1	–	resistance
	–	upregulating a subset of S100 proteins and triggering Src kinase signalling	vascular leakiness
	miR-23a, miR-105	targeting ZO-1	vascular leakiness
	miR-9	–	shift of normal FBs into tFBs
	HER2	competitive inhibition	Trastuzumab resistance
PDAC	circ-PDE8A	miR-338/MACC1/MET	tumour invasion
Lung cancer	miR-23a, miR-105	targeting ZO-1	vascular leakiness
Myeloma	syndecan-1, VEGF, HGF	–	angiogenesis
	EGFR	MAPK, AKT	angiogenesis
UBC	LncRNA-HOTAIR	–	EMT
	circPRMT5	miR-30c “sponge”	EMT
Gastric cancer	LncRNA-ZFAS1	–	EMT
Pancreatic cancer	SELPLG	–	coagulation and metastasis
HNSCC	PD-L1	competitive inhibition	immunosuppression, tumour progression
Prostate cancer	PD-L1	competitive inhibition	immunosuppression
B-cell lymphoma	CD20	competitive inhibition	rituximab resistance

The physiological and pathological study of tumours can be mainly categorised into two levels: (1) single molecule studies, which study a specific molecule in EVs and its role; and (2) functional observational studies, which determine the changes and possible roles of EVs and their cargo in the TME. There is a very large gap between the functional observational studies and the single molecule studies, as well as between in vivo and in vitro studies.

EVs through their cargo play a variety of different roles, but it is important to determine which of these play primary or secondary roles, to what percentage this function is carried out, and whether there is synergy or antagonism between these molecules or EVs. Current research is not sufficient to provide satisfactory answers. There is also a gap between molecules or single source EVs and the TME. This is because the TME is complex, containing many cells and molecules carrying out different functions. Rather than a single molecule or single source of EVs contributing to their functions, there is an interplay between various molecules and all sources of EVs.

Moreover, cellular communication has not been well studied. The interaction between cells is bidirectional, and tumour cells and non-malignant cells in the body continuously interact with each other in dynamic equilibrium to form the TME. Many in vitro experiments are carried out using EVs from one type of cell to stimulate another type of cell. The effect of EVs on cells in the TME is not a simple and direct effect, but rather an effect similar to the “iterative effect”, such that the influence of T-EVs on non-malignant cells can also affect the content of nm-EVs; and the influence of affected nm-EVs on tumours and T-EVs is changed. When this process is continuously repeated, the features of EVs will completely deviate from the simple in vitro model. Although it is not clear what kind of new model will be most appropriate, organ-on-a-chip may be a more appropriate option for future research [238].

Minimal Information for Studies of Extracellular Vesicles 2018 (MISEV2018) endorses EVs as the standard terminology for vesicles which are released naturally by the cell and enclosed within a lipid bilayer without replication capacity (without a functional nucleus) [239]. Over the years, many enlightening studies have been carried out in this field of EVs, but there are still fundamental questions that need to be resolved in future. One of the biggest problems is around the “EV subtypes”. Since consensus has not yet been reached on specific markers of EV subtypes (for instance, endosome-origin “exosomes”, and plasma membrane-origin “MVs”) [239–242], there are still great difficulties in attributing a specific EV to a specific biogenic mechanism, unless an EV is observed in the process of release by a live imaging system [239]. Therefore, although many authors have classified EVs into subtypes based on particle size and density, as long as a set identification system/principle, with reliable specific markers of subcellular origin, cannot be established, the terminology of EV subtypes should be avoided [239]. Thus, establishment of reliable specific markers of subcellular origin is an important scientific issue, to which researchers in the EV field should pay attention.

Finding discriminating markers for T-EVs versus normal stromal EVs has been an important research direction in this field for a long time. Although a number of markers on EVs have been identified for a particular type of tumour (for example: EpCAM for ovarian cancer; VLA-4, TYRP2 and MET for melanoma; MIF for PDAC), so far, no universal markers have been found. If one or more universal/general markers, which can cover various kinds of tumours not just one or a few, can be found on T-EVs, then the use of T-EVs for tumour diagnosis will become even more meaningful. If no universal markers can be found, then compromise tactics will need to be used. EVs from various tumour sources can be analysed, and the data can be uploaded to a database. Through bioinformatics technology, a minimal set of genes could be found, which can cover as many tumours as possible, and the database should be updated as often as possible.

New evidence shows that EV-circRNAs could possess important biological functions in different pathological and physiological procedures. EV-circRNAs are confirmed to be extremely stable [243]. Furthermore, genome-wide studies have determined that the quantity and proportion of circular-to-linear splicing is a minimum of two to six times greater in EVs than in producer cells. There are also over 1000 distinctive circRNA candidates available in individual serum EVs [12]. However, too few studies have investigated the specific mechanism of EV-circRNAs in the TME. Moreover, the stability of EV-circRNAs gives them excellent potential for carrying out EV-based liquid biopsy. This could be a good avenue for follow-up research, and could generate new ideas to advance the understanding of diseases, as well as new diagnosis and treatment methods.

Liquid biopsy is based on the detection and analysis of biomarkers (for instance, circulating tumour cells (CTCs), cell-free nucleic acids (cfNAs), and EVs) in readily-available body fluids such as peripheral blood [244–246]. The first step of major liquid biopsy approaches is isolation and enrichment of targets [246], and it is important to establish a reliable system.

Analysis of EVs is a promising potential new star in the field of liquid biopsy, and an approach that has attracted more and more attention in recent years is single-EV analysis, which is of crucial significance for the precise analysis and diagnosis of diseases [246]. A multiplexed fluorescent imaging system has been introduced by Lee and colleagues for the detection and analysis of multiplex markers on a single EV, and this technology can analyse up to 11 different markers [247]. Microfluidic technology has also played an important role in promoting the development of this field. A nano-interfaced microfluidic EV (nano-IMEX) platform was reported by Zhang and colleagues, and this platform has the capacity to distinguish ovarian cancer patients from controls by detecting un-processed minimal volume (2 μL) plasma samples [248]. Consequently, when combined with nanotechnology and microfluidic technology, liquid biopsy is likely to bring many new advances in the field of EVs, although the problem that needs to be solved to enable these future developments remains identification of discriminating markers and stable and reliable methods of separation, extraction and purification.

EVs are involved in various pathophysiological conditions such as development and progression of disease [35, 249]. Several recent studies have identified specific inhibitors which block the predominant EV subpopulations (for example, GW4869 for Exos, or Y27632 for MVs) [250]. However, even if some of these inhibitors, which have already been formulated and used as therapeutic agents, have proven to be reliable and robust and have reproducible inhibitory effects on the release of EVs, the side-effects must also be considered, for instance, the side-effects of imipramine include immune suppression and infections, nausea, vomiting, dizziness, tiredness, disorientation and low blood pressure, while the side-effects of pantetheine include impaired blood clotting, nausea, and diarrhoea [250]. The ultimate goal would be to selectively and effectively influence EVs involved in pathological processes but not those performing necessary physiological roles, but so far this goal has not been achieved [250]. Based on what has been achieved so far, tactics to selectively target delivery of these drugs to malignant tumours but not normal tissues may be a straightforward and feasible solution.

Although many of the EV-based therapeutic approaches performed well in pre-clinical models and phase I clinical trials, they exhibited many notable issues in subsequent phase II clinical trials. In patients with advanced non-small cell lung carcinomas, interferon-γ (IFN-γ)-DC-EV treatment ceased to be effective, revealed by long-term clinical observation [251]. Therefore, ways in which basic research can be progressed towards clinical application is also an issue to be addressed in future research.

On account of their good stability, long circulating half-life and relatively good bio-safety, EVs are considered to be potential drug delivery systems with high delivery efficiency and low toxicity [252]. Moreover, studies have also shown that EVs have a unique “homing” ability (the capability to target the cell type similar to their source cells) [252, 253]. Studies have shown that EVs actively target a specific cell type through a variety of mechanisms especially receptor–ligand recognition [254], and thus, by engineering EVs loaded with specific ligands (including antibodies, peptides, and aptamers) onto their surfaces, the targeting ability of EVs can be changed so that they target the cells that need them for functional intervention on specific cells [255, 256].

Aptamers are RNAs or single-stranded DNAs (ssDNAs) folded into particular 3D structures with high specificity and affinity through a similar mechanism to antigen–antibody binding [257]. Aptamers are a new favourite due to their relatively high stability, minimal toxicity, lack of immunogenicity, and superb tissue penetration [258]. Systematic evolution of ligands by exponential enrichment (SELEX), an in vitro aptamer selection and screening process, comes with a very powerful tool to select and isolate organ-specific aptamers [259]. Combining engineered EVs and aptamers can enable development of better EV-based targeted drug delivery systems. In a similar way to the use of viruses and liposomes for transfection, as are commonly used in biological research, “click chemistry” could be used to assemble ligands onto the surface of EVs [260, 261]. There are also many techniques for loading small molecule compounds, nucleic acids and proteins into EVs [36]. Taken together, these advances in bio-engineering of EVs will bring very promising new treatment tactics to advance the treatment of malignant tumours.

CRISPR/Cas9 treatment through EVs is also a very promising research prospect. At present, this technology is mainly used as a research tool. The most typical application of this treatment is to confirm the spread of EVs as reporters. It would be interesting to compare EV-based CRISPR/Cas9 with known methods using CRISPR/Cas9 for gene therapy, in addition to determining the differences in the scope of application, treatment effects, and the advantages and disadvantages of this approach.

## Conclusion

EVs function as a transport medium for various molecules in the TME, and therefore have a variety of potential uses in the diagnosis and treatment of cancer. EVs also participate in the progression of various processes involved in malignant tumour development. Tumour cells and non-malignant cells typically communicate with each other, together determining the progress of the disease. Although T-EVs are known for orchestrating tumour advancement via systemic pathways, nmEVs also contribute substantially to malignant tumour development.

In this review, we have summarised the features of both T-EVs and nmEVs, and their roles in tumour progression, metastasis, and EV-mediated chemoresistance in the TME. This review discusses recent and current research regarding the clinical applications of EVs, the findings of these studies, and how this information can be used to repurpose EVs as a therapeutic tool. This sound and current overview of the present research, questions to be addressed, and potential directions for future research in the field makes a significant contribution to the literature.

## Data Availability

Please contact author for data requests.

## Abbreviations

EVs: Extracellular vesicles
T-EVs: Tumour-derived EVs
MSC-EVs: MSC-derived EVs
Mφ-EVs: Mφ-derived EVs
tFB-EVs: tFB-derived EVs
DC-EVs: DC-derived EVs
nmEVs: EVs derived from non-malignant cells
mRNAs: messenger RNAs
miRNAs: microRNAs
LncRNAs: Long non-coding RNAs
circRNAs: circular RNAs
ssDNA: single-stranded DNA
dsDNA: double-stranded DNA
PMN: Pre-metastatic niche
ceRNA: Competing endogenous RNAs
EMT: Epithelial–mesenchymal transition
ECM: Extracellular matrix
FBs: Fibroblasts
ECs: Endothelial cells
MSCs: Mesenchymal stem cells
EpiCs: Epithelial cells
Mφs: Macrophagocytes
NK: Natural killer
Treg: T regulatory
DCs: Dendritic cells
MDSCs: Myeloid-derived suppressor cells
HSCs: Haematopoietic stem cells
PBLCs: Peripheral blood lymphocytes
tFBs: Tumour-associated FBs
CAFs: Cancer-associated fibroblasts
t-Mφs: Tumour-associated Mφs
M1-Mφs: M1 subtype Mφs
M2-Mφs: M2 subtype Mφs
Ad-MSCs: Adipose tissue-derived MSCs
MFBs: Myofibroblasts
PDAC: Pancreatic ductal adenocarcinoma
HCC: Hepatocellular carcinoma
HNSCC: Head and neck squamous cell carcinoma
RCC: Renal cell cancer
PC: Pancreatic cancer
UBC: Urothelial bladder cancer
CRC: Colorectal cancer
GC: Gastric cancer
Exos: Exosomes
MVs: Microvesicles
MVBs: Multi-vesicular bodies
ILVs: Intraluminal vesicles
HGF: Hepatocyte growth factor
PDGFR: Platelet-derived growth factor receptor-beta
TFs: Tissue factors
VEGF: Vascular endothelial growth factor
TNF-α: Tumour necrosis factor α
TGF-β: Transforming growth factor beta
APAF1: Apoptotic peptidase activating factor
PGE2: Prostaglandin E2
SDF-1: Stromal cell-derived factor 1
TYRP2: Tyrosinase-related protein-2
VLA-4: Very late antigen 4
IL-6: Interleukin-6
MMP: Matrix metalloproteinase
Cdh17: Cadherin-17
MICA: MHC class 1 related chain ligand A
EGFR: Epidermal growth factor receptor
EGFRvIII: EGFR variant III
ER: Estrogen receptor
Fas-L: Fas ligand
SELPLG: P-selectin glycoprotein ligand 1
TLR: Toll-like receptor
CCL5: C-C motif chemokine ligand 5
TCR: T cell receptor
EpCAM: Epithelial cell adhesion molecule
MAPK: Mitogen-activated protein kinase
PKB: Protein kinase B
SOCS5: Suppressor of cytokine signalling 5
STAT3: Signal transducer and activator of the transcription 3
PCP: Planar cell polarity
SOCS: Suppressor of cytokine signalling
DOX: Doxorubicin
EV-DOX: EVs carrying doxorubicin
TME: Tumour microenvironment
Met-high: High levels of Met
TNM: Tumour node metastasis
KD: Knockdown
WAT: White adipose tissue
HCV: Hepatitis C virus
mAB: monoclonal antibody
circ-DB: circ-de-ubiquitination
ADAPT™: Adaptive Dialysis-like Affinity Platform Technology
